# P-31. Implementation of a Multi-Faceted Intervention to Improve Vaccination Rates in Older Adults

**DOI:** 10.1093/ofid/ofae631.238

**Published:** 2025-01-29

**Authors:** Laurie Archbald-Pannone, Angie D Settle, Leah Molloy, Laura Simone, Chris Napolitan, Jeffrey D Carter, Jacqueline Maytorena, Kelly E Pillinger

**Affiliations:** University of Virginia School of Medicine, Charlottesville, Virginia; West Virginia Health Right, Inc., Charleston, West Virginia; PRIME Education, Brighton, Michigan; PRIME Education, LLC, Fort Lauderdale, Florida; PRIME Education, Brighton, Michigan; PRIME Education, LLC, Fort Lauderdale, Florida; PRIME Education, Brighton, Michigan; PRIME Education, Brighton, Michigan

## Abstract

**Background:**

Older adults are at high risk for poor outcomes from infectious diseases, yet rates of recommended vaccinations remain low among this age group. This quality improvement initiative aimed to increase vaccination engagement among older adults through patient-provider collaborative learning sessions (CLS) and use of a customized shared decision-making (SDM) tool.

**Figure:**
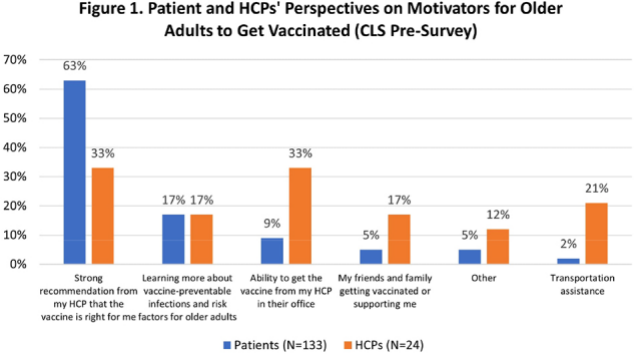

**Methods:**

From 8/23 – 1/24, the CLS and SDM tools were implemented in 6 primary care/geriatric clinics with surveys administered before and after. Healthcare professionals (HCPs) participated in audit-feedback (A/F) sessions to create action plans to improve their clinic’s vaccination rates. Follow-up patient and HCP surveys were administered 4-6 weeks and 90 days later, respectively.

**Results:**

The top reported barriers to vaccination amongst the 133 patients who participated in the CLS were knowing which vaccinations were needed (57%) and paying for vaccines (54%). Following the CLS, patients’ knowledge about hospitalization due to RSV significantly increased from 40% (53/133) to 65% (80/123) (P < 0.001). Additionally, 59% (73/123) of patients stated they now planned to get recommended vaccines. Most patients (67%) listed a strong recommendation from a HCP as the best motivator to getting vaccinated, however, only 33% (8/24) of HCPs perceived that would be important (Figure 1).

Of the 72 patients who used the SDM tool and completed both surveys, only 40% reported being up-to-date on all recommended vaccines and 76% had not received a single dose of the shingles vaccine; 65% had not regularly discussed barriers to vaccination with their HCP. However, after using the SDM tool, 81% of patients reported they discussed their vaccination concerns more or much more than previous visits. Patients were more willing to get vaccinated following use of the SDM tool (72% to 89%, P = 0.01). Of the 14 HCPs who completed follow-up surveys, 57% reported increased vaccination rates following CLS and SDM implementation, with use of SDM (50%) and incorporating presumptive language into vaccine discussions (43%) as the top strategies employed.

**Conclusion:**

Use of multi-faceted strategies to engage older adult patients in vaccine decision-making increased clinic-reported vaccine rates and patient-reported willingness to get vaccinated.

**Disclosures:**

**Laurie Archbald-Pannone, MD, MPH**, prime, inc: Advisor/Consultant **Kelly E. Pillinger, PharmD**, AHFS: Contractor

